# Supplementary material for: Myo9b mutations are associated with altered dendritic cell functions and increased susceptibility to autoimmune diabetes onset
Source: Nat Commun. 2023 Sep 25;14:5977. doi: 10.1038/s41467-023-41534-w (PMC10519942; doi:10.1038/s41467-023-41534-w)
Supplement: Supplementary file 1 — Supplementary Information [file 41467_2023_41534_MOESM1_ESM.pdf]

Supplementary Information

Myo9b mutations are associated with altered dendritic cell functions and increased susceptibility to autoimmune diabetes onset

Supplementary Figures

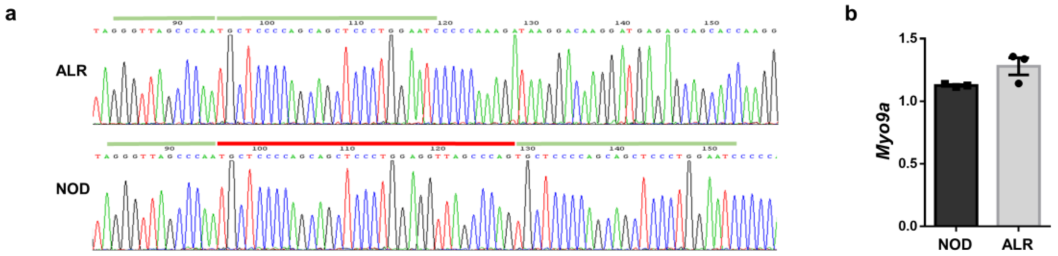

Supplementary Figure 1. Comparison of *Myo9b* DNA sequences and *Myo9a* mRNA levels between NOD and ALR mice. (a) Comparison of *Myo9b* DNA sequences between NOD and ALR mice. (b) mRNA levels of *Myo9a* in NOD and ALR BMDCs as measured by real-time PCR. Data are represented as mean ± SEM of 3 independent biological replicates. Statistical significance was determined by unpaired two-sided Student's *t* test.

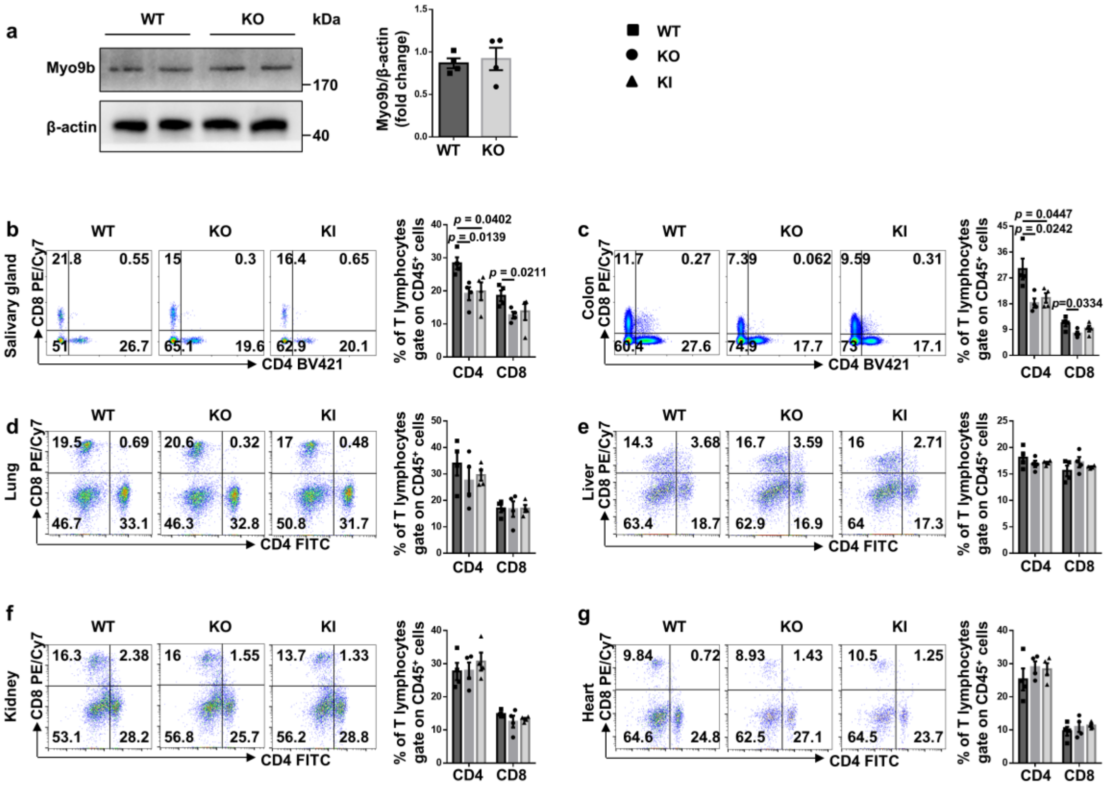

Supplementary Figure 2. Quantification of T lymphocyte percentages in indicated tissues. (a) Representative western blot analysis and quantification of Myo9b levels in CD4⁺ T cells from WT and KO mice. (b-g) Representative flow cytometry plots and percentages of T cells among total CD45⁺ immune cells in salivary gland, colon, lung, liver, kidney, and heart from 12-week-old WT, KO and KI mice. *n* = 4 per group. Values are presented as mean ± SEM. Statistical difference in (a) was analyzed using unpaired two-sided Student's *t* test; and in (b-g) was determined by one-way ANOVA.

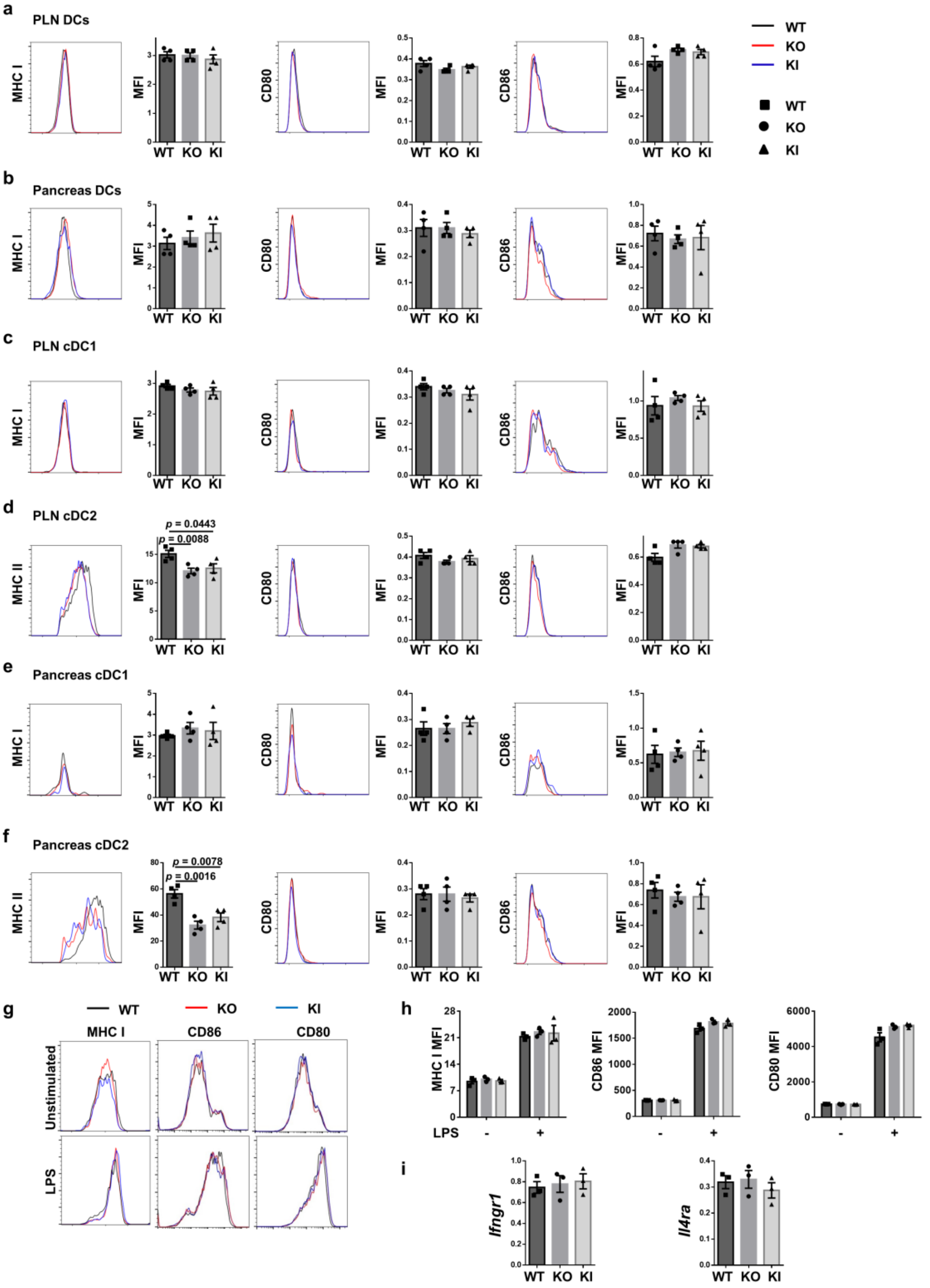

Supplementary Figure 3. Effect of ALR *Myo9b* KI and *Myo9b* deficiency on expression of MHC and co-stimulatory molecules in DCs. (a-f) PLN and pancreas cells from 10- to 12-week-old WT, KO, and KI mice were harvested and subjected to flow cytometry analysis (*n* = 4 per group). (a, b) Flow cytometry analysis of MHC I, CD80, and CD86 expression in CD11c^{+}MHC II^{+} cells of PLNs (a) and pancreas (b). (c,

d) Flow cytometry analysis of MHC molecules, CD80, and CD86 expression in cDC1 (c) and cDC2 (d) of PLNs. (e, f) Flow cytometry analysis of MHC molecules, CD80, and CD86 expression in cDC1 (e) and cDC2 (f) of pancreas. (g, h) Representative histograms (g) and quantitative data (h) of MHC I, CD86, and CD80 expression in WT, KO, and KI BMDCs treated with vehicle or LPS for 24 h. (i) RT-PCR analysis of relative mRNA expression of *Ifngr1* and *Il4ra* in BMDCs stimulated with LPS for 8 h. Data were collected from three independent experiments (g-i). Values are presented as mean ± SEM. Significance was determined by one-way ANOVA.

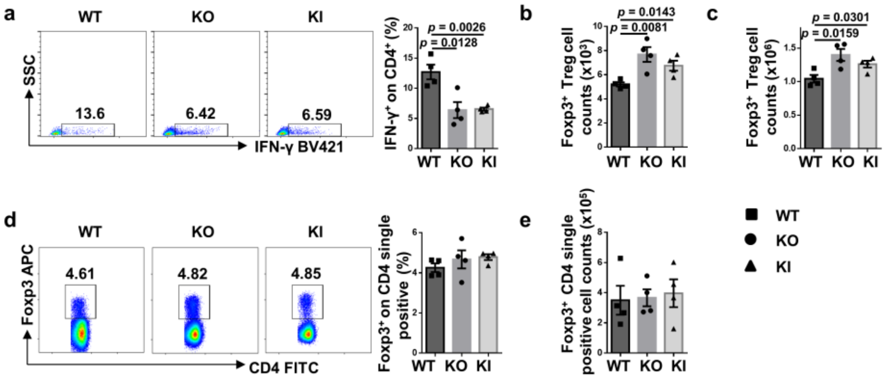

Supplementary Figure 4. Analysis of peripheral and thymic Treg cells in WT, KO, and KI mice. (a) Representative FACS plots and frequencies of Th1 cells in the PLNs of 16-week-old WT, KO, and KI mice. (b, c) Quantification of Foxp3^{+} Treg cells in the PLNs (b) and spleen (c) of 10- to 12-week-old WT, KO, and KI mice. (d) Representative flow cytometry plots and frequencies of thymic Treg cells among CD4 single positive cells in 10- to 12-week-old WT, KO, and KI mice. (e) Quantification of thymic Foxp3^{+} CD4 single positive cells in 10- to 12-week-old WT, KO, and KI mice. n = 4 in each study group (a-e). Values are expressed as mean ± SEM. Statistical difference was determined by one-way ANOVA.

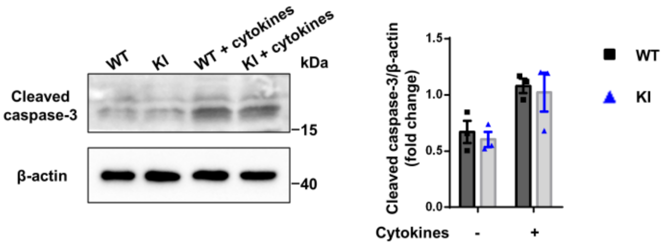

Supplementary Figure 5. Representative western blot analysis and quantification of cleaved caspase-3 levels in pancreatic islets with or without proinflammatory cytokines (IL-1β + TNF-α + IFN-γ) stimulation for 24 h. Data were collected from three independent experiments. Values are presented as mean ± SEM, and unpaired two-sided Student's *t* test was employed for data analysis.

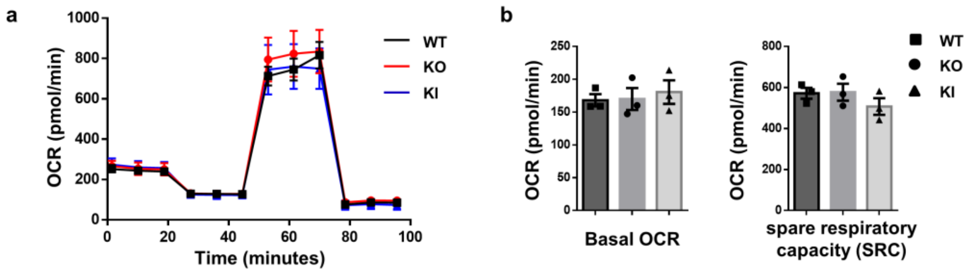

Supplementary Figure 6. (a) OCR of BMDCs derived from WT, KO, and KI mice following LPS stimulation for 24 h, which was measured before and after sequential treatment with oligomycin, FCCP, and rotenone plus antimycin A. (b) Accordingly, basal OCR and the spare respiratory capacity (SRC) were shown. Data derived from 3 independent experiments are presented as mean ± SEM. Significance was determined by one-way ANOVA.

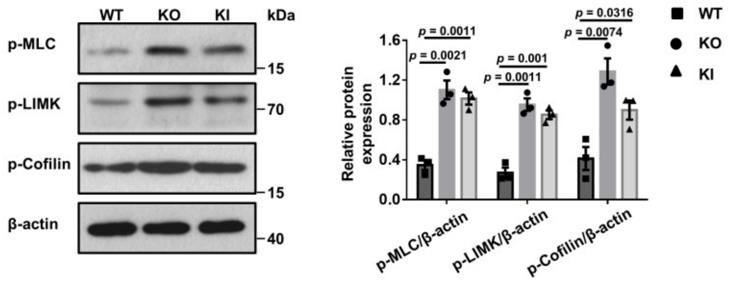

Supplementary Figure 7. Expression levels of p-LIMK, p-Cofilin, and p-MLC in BMDCs as detected by Western blot. Data were collected from three independent experiments. Values are presented as mean ± SEM and one-way ANOVA was used for data analysis.

a Rs766200985 A>C

Subjects	*N*	Genotyping distribution (AA / AC / CC)	MAF
T1D	1298	1292 / 6 / 0	0.23%
Control	2936	2926 / 10 / 0	0.17%

OR = 1.359, *p* = 0.590, 95% CI = 0.405-4.137

b Rs776331004 G>A

Subjects	*N*	Genotyping distribution (GG / GA / AA)	MAF
T1D	1298	1298 / 0 / 0	0%
Control	2936	2934 / 2 / 0	0.034%

OR = 0, *p* = 1, 95% CI = 0-12.049

Supplementary Figure 8. Genotyping results for Rs766200985 (a) and Rs776331004 (b) in T1D cases and healthy controls. Statistical differences were assessed using Fisher's exact test.

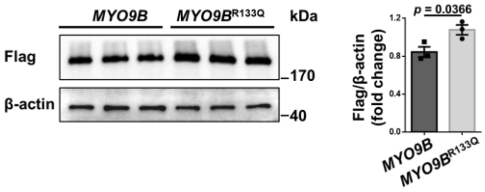

Supplementary Figure 9. MYO9B expression in *Myo9b*$^{-/-}$ mouse DCs transduced with *MYO9B*^{WT} or *MYO9B*^{R133Q} virus. Values are presented as mean ± SEM of three independent experiments. Significance was determined by unpaired two-sided Student's *t* test.

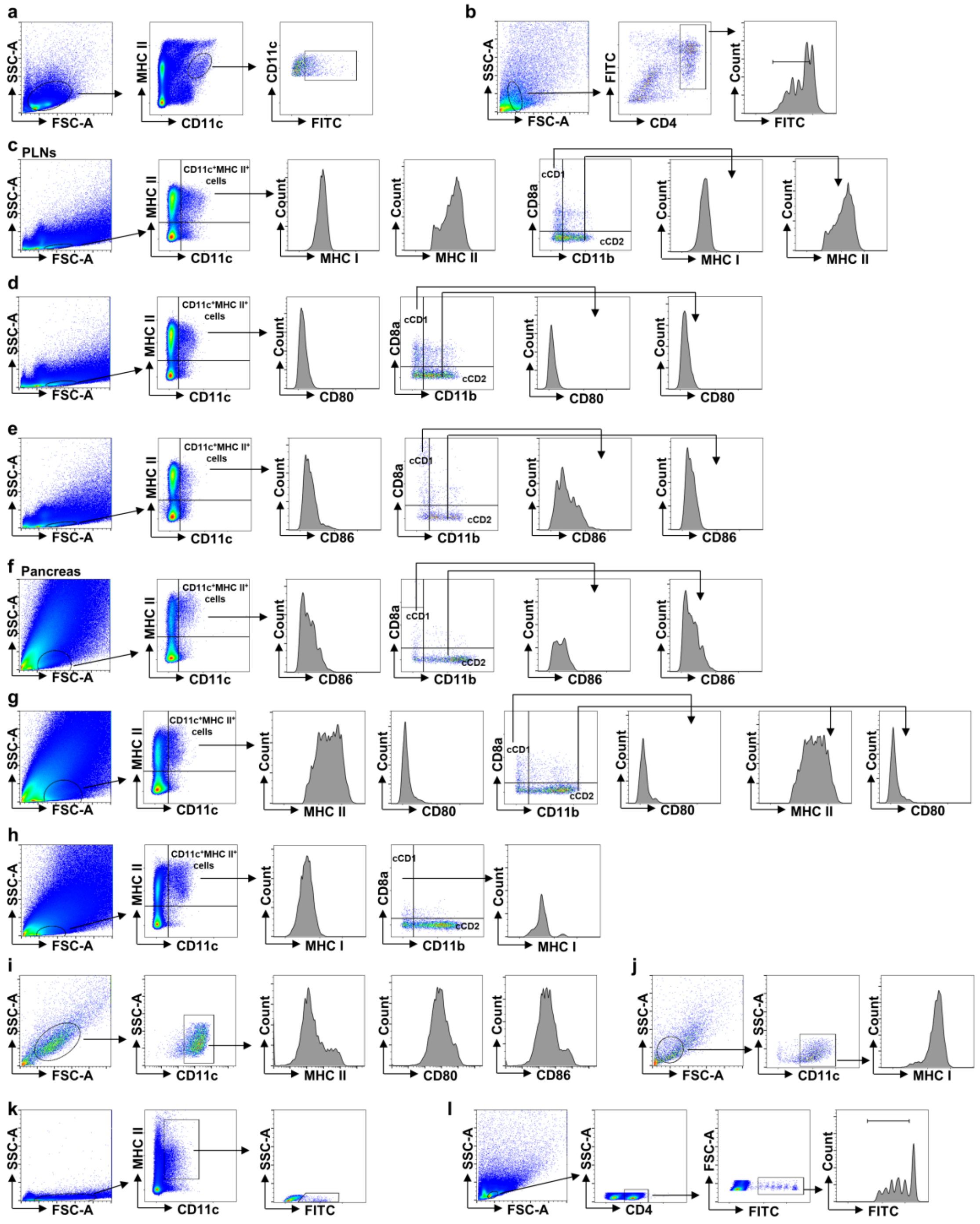

Supplementary Figure 10. Gating strategy used for flow cytometry analysis. Gating strategies for Fig. 1c (a); Fig. 1d, 4f, 8g, 10e, 10k (b); Fig.3a, 3c, Supplementary Fig.3a, 3c, 3d (c); Fig.3e, Supplementary Fig.3a, 3c, 3d (d); Supplementary Fig.3a, 3c, 3d (e); Fig. 3b, Supplementary Fig.3b, 3e, 3f (f); Fig. 3d, 3f, Supplementary Fig.3b, 3e, 3f (g); Supplementary Fig.3b, 3e, 3f (h); Fig. 4a, 4b, Fig. 8e, Fig. 10c, Supplementary Fig.3g (i); Supplementary Fig.3g (j); Fig. 4e (k); Fig. 4g (l).

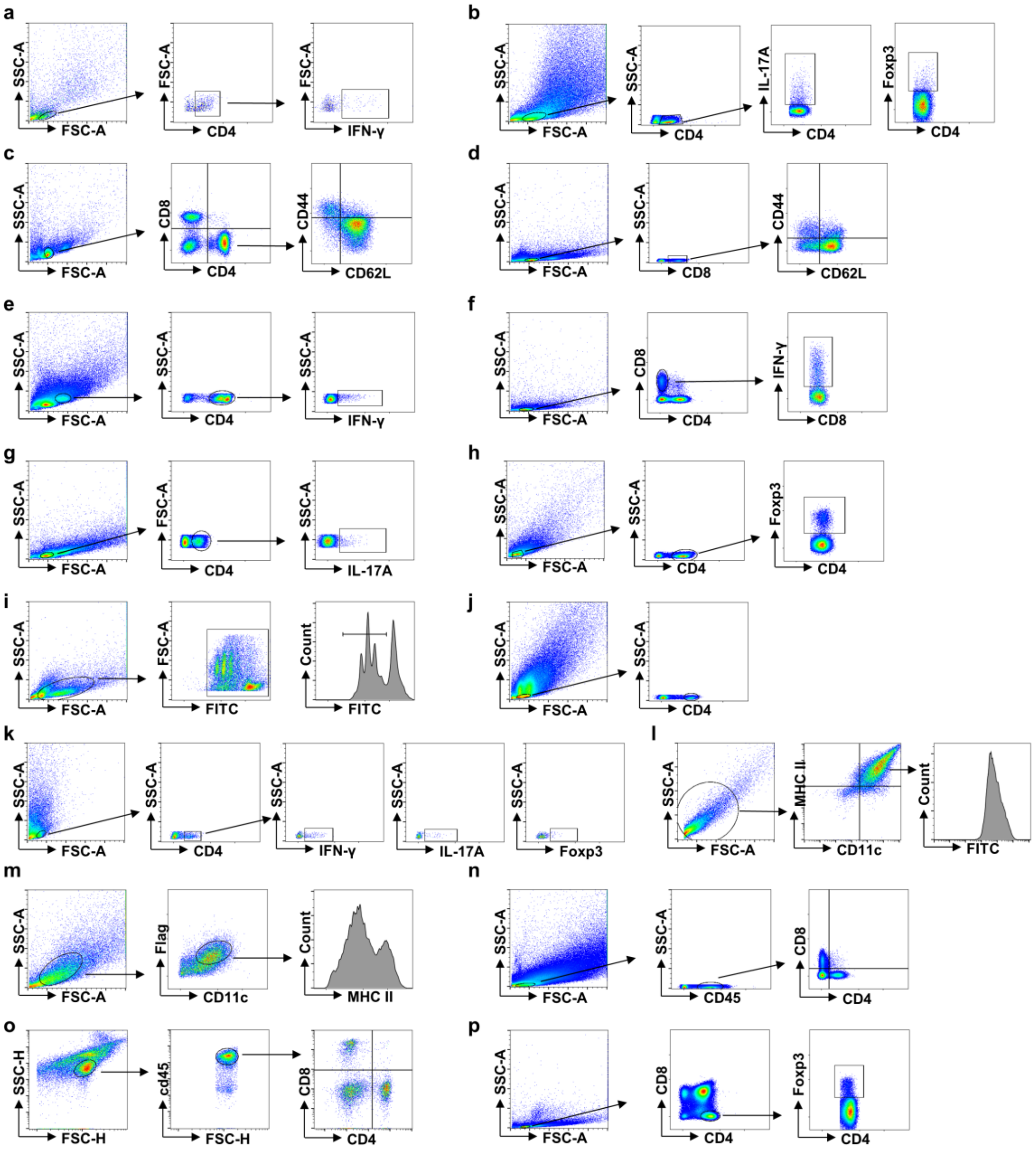

Supplementary Figure 11. Gating strategy used for flow cytometry analysis. Gating strategies for Fig. 4h (a); Fig. 4i, 4j (b); Fig. 5a, 5b, 5h, 5i (c); Fig. 5c, 5j (d); Fig. 5d, 5k, Supplementary Fig. 4a (e); Fig. 5e, 5l (f); Fig. 5f, 5m (g); Fig. 5g, 5n (h); Fig. 5o (i); Fig. 6d (j); Fig. 6e-g (k); Fig. 7f (l); Fig. 10h (m); Supplementary Fig. 2b, 2c (n); Supplementary Fig. 2d-g (o); Supplementary Fig. 4d (p).

Supplementary Tables

Supplementary Table 1. The frequencies and annotations of identified *MYO9B* variants in 260 T1D cases and 240 healthy controls.

Pos	Ref	Alt	Func	normal_Hom	normal_Het	normal_NoVar	T1DM_Hom	T1DM_Het	T1DM_NoVar
17212907	C	T	missense_variant	0	0	240	0	1	259
17263461	G	A	missense_variant	0	0	240	0	1	259
17263509	G	A	missense_variant	0	0	240	0	1	259
17264827	G	A	missense_variant	0	0	240	0	1	259
17291846	T	G	missense_variant	0	0	240	0	1	259
17283156	G	A	missense_variant	0	0	240	0	1	259
17305429	G	A	missense_variant	0	0	240	0	1	259
17305466	C	T	missense_variant	0	0	240	0	1	259
17305559	G	A	missense_variant	0	0	240	0	1	259
17306038	G	A	missense_variant	0	0	240	0	1	259
17303796	G	A	missense_variant	0	0	240	0	1	259
17316944	G	A	missense_variant	0	0	240	0	1	259
17322769	G	A	missense_variant	0	0	240	0	1	259
17303807	C	T	missense_variant	0	1	239	0	0	260
17309021	G	A	missense_variant	0	1	239	0	0	260
17313005	G	A	missense_variant	0	1	239	0	0	260
17313092	A	G	missense_variant	0	1	239	0	0	260
17313676	G	A	missens	0	1	239	0	0	260

			e_variant						
17314011	C	T	missense_variant	0	1	239	0	0	260
17291849	T	C	missense_variant+splice_region_variant	0	1	239	0	0	260
17298786	C	T	missense_variant	0	1	239	0	0	260
17212612	G	A	missense_variant	0	1	239	0	0	260
17212865	A	G	missense_variant	0	1	239	0	0	260
17265138	T	C	missense_variant	0	1	239	0	0	260
17317179	G	A	missense_variant	0	1	239	0	0	260
17317548	A	G	missense_variant	0	1	239	0	0	260
17322565	G	A	missense_variant	0	1	239	0	0	260
17322935	G	A	missense_variant	0	1	239	0	0	260
17317529	G	A	missense_variant	0	1	239	0	1	259
17212518	G	A	5_prime_UTR_variant	0	0	240	0	2	258
17278758	A	C	missense_variant	0	0	240	0	2	258
17212925	G	A	missense_variant	0	2	238	0	0	260
17303595	C	T	missense_variant	0	2	238	0	2	258
17316782	T	C	missense_variant	139	93	8	156	88	16
17303774	T	G/A	missense_variant	135	96	9	154	90	16

Pos, position. Ref, Reference allele. Alt, Alternate allele

Supplementary Table 2. Minor Allele Frequencies

MYO9B Locus T1D SNPs tested		Rs764932023
		G/G
		G/A
		A/A
Ref		G
Alt		A
Observed Alt in our result	Normal(n=2936)	0.085%
	T1D(n=1298)	0.54%
Global (n=247774) (gnomAD – Exomes)		0.0121%
European (n=133218) (gnomAD – Exomes)		0.0023%
American (n=13664)		0
African (n=42054) (gnomAD – Genomes)		0.002%
Asian (n=48544) (gnomAD – Exomes)		0.047%
Japanese (n=16756) （8.3KJPN）		0.143%
Korean (n= 1832) Korean Genome Project		0.11%

Supplementary Table 3. Primer sequences for real-time PCR

Gene	Forward (5'-3')	Reverse (5'-3')
**Mouse *Myo9a***	TGTCCAAATCATAGCAAGTGCC	CTGCCGAAATTCTTTCAGGGC
**Mouse *Il12***	CTCCTAAACCACCTCAGTTTG	CAGGAATAATGTTTCAGTTTTTC
**Mouse *Il6***	ATGGATGCTACCAAACTGGAT	TGAAGGACTCTGGCTTTGTCT
**Mouse *Tnf***	ACTGAACTTCGGGGTGATCG	GGCTACAGGCTTGTCACTCG
**Mouse *Ifngr1***	TACAGGTAAAGGTGTATTCGGGT	ACCGTGCATAGTCAGATTCTTTT
**Mouse *Il4ra***	AACCTGTACCCATCGAACAAC	GCCGTATAGTAGACCCCTGAC
**Mouse *Actb***	AGAGGGAAATCGTGCGTGAC	CAATAGTGATGACCTGGCCGT
**Human *IL6***	ACTCACCTCTTCAGAACGAATTG	CCATCTTTGGAAGGTTCAGGTTG
**Human *IL12***	CCTTGCACTTCTGAAGAGATTGA	ACAGGGCCATCATAAAAGAGGT
**Human *TNF***	CCTCTCTCTAATCAGCCCTCTG	GAGGACCTGGGAGTAGATGAG
**Human *ACTB***	CATGTACGTTGCTATCCAGGC	CTCCTTAATGTCACGCACGAT

Supplementary Table 4. Clinical characteristics of the subjects

	healthy control	T1D case
**Number**	2936	1298
**Age (years)**	28.78±0.28	26.26±0.42
**Sex (M/F)**	1547/1389	619/679
**BMI**	22.75±0.96	19.19±0.11
**HbA1c (%)**	-	9.89±0.10
**HbA1c (mmol/mol)**	-	
**FPG (mmol/L)**	4.85±0.02	12.33±0.27
**2HG (mmol/L)**	5.64±0.05	15.67±0.31
**FCP (ng/ml)**	-	0.20±0.01
**2HCP (ng/ml)**	-	0.34±0.02
**Diabetes duration (months)**	-	37.94±1.72
**Number of subjects with IAA$^+$**	-	599
**Number of subjects with GADA65$^+$**	-	932
**Number of subjects with ZnT8$^+$**	-	282

Supplementary Table 5. The sequences of primers and probes for genotyping

Gene	Primer (5'-3')	Probe
**rs776331004**	Forward CTGCCTGCCTCTTTCACACT<br>Reverse ACTTCCTCCCACCATTCTGAC	VIC-CGGCCGCCTGGCTT-BHQ1<br>FAM-CCTGCCGGCTGCCTG-BHQ1
**rs764932023**	Forward CTGACTGTTACCTATTAAAACTTTAGAG<br>Reverse GAATGTCTGAATTATTATTCTAGGTTC	VIC-CAGCCACCCGGCGC-BHQ1<br>FAM-AGCCACCCAGCGCCT-BHQ1
**rs766200985**	Forward GTCTGTGTAGCCGATGTTGTG<br>Reverse AAGAAACTTACCGACCCCGC	VIC-CCCTGATATTCCTCC-MGB<br>FAM- CTGATAGTCCTCCTG-MGB
