# Supplementary material for: Myo9b mutations are associated with altered dendritic cell functions and increased susceptibility to autoimmune diabetes onset
Source: Nat Commun. 2023 Sep 25;14:5977. doi: 10.1038/s41467-023-41534-w (PMC10519942; doi:10.1038/s41467-023-41534-w)
Supplement: Supplementary file 3 — Reporting Summary [file 41467_2023_41534_MOESM3_ESM.pdf]

nature portfolio

Corresponding author(s): Jinhua Yan, Zhiguang Zhou and Cong-Yi Wang

Last updated by author(s): Aug 15, 2023

Reporting Summary

Nature Portfolio wishes to improve the reproducibility of the work that we publish. This form provides structure for consistency and transparency in reporting. For further information on Nature Portfolio policies, see our Editorial Policies and the Editorial Policy Checklist.

Statistics

For all statistical analyses, confirm that the following items are present in the figure legend, table legend, main text, or Methods section.

n/a	Confirmed	
☐	☒	The exact sample size (*n*) for each experimental group/condition, given as a discrete number and unit of measurement
☐	☒	A statement on whether measurements were taken from distinct samples or whether the same sample was measured repeatedly
☐	☒	The statistical test(s) used AND whether they are one- or two-sided<br>*Only common tests should be described solely by name; describe more complex techniques in the Methods section.*
☒	☐	A description of all covariates tested
☐	☒	A description of any assumptions or corrections, such as tests of normality and adjustment for multiple comparisons
☐	☒	A full description of the statistical parameters including central tendency (e.g. means) or other basic estimates (e.g. regression coefficient) AND variation (e.g. standard deviation) or associated estimates of uncertainty (e.g. confidence intervals)
☐	☒	For null hypothesis testing, the test statistic (e.g. *F*, *t*, *r*) with confidence intervals, effect sizes, degrees of freedom and *P* value noted<br>*Give P values as exact values whenever suitable.*
☒	☐	For Bayesian analysis, information on the choice of priors and Markov chain Monte Carlo settings
☒	☐	For hierarchical and complex designs, identification of the appropriate level for tests and full reporting of outcomes
☒	☐	Estimates of effect sizes (e.g. Cohen's *d*, Pearson's *r*), indicating how they were calculated

Our web collection on statistics for biologists contains articles on many of the points above.

Software and code

Policy information about availability of computer code

Data collection	Blood glucose was monitored using an Accu-Check Advantage glucometer (Roche Diagnostics, Indianapolis, IN, USA).<br>OLYMPUS Upright microscope BX53 was used to acquire microscopy images.<br>MACSQuantTM was applied to acquire flow cytometry data.<br>Seahorse XFe24 analyzer was used to measure the extracellular acidification rate and oxygen-consumption rate.<br>Total RNA libraries were sequenced on an Illumina HiSeq 2500 instrument.<br>Genotyping for the Myo9b variants was performed using the TaqmanTM 7900HT Sequence Detection System.
Data analysis	FlowJo software v10.5.3 was used to analyze flow cytometry data. ImageJ 1.46r software was used to analyze western blot data and cell migration tracks. Seahorse metabolic data were analyzed using the XFe Wave software (Agilent Technologies, Santa Clara, CA, USA). The fragments per kilobase of exon per million fragments mapped (FPKM) was calculated using the Bowtie2 software. Differential expression analysis was performed using DESeq2 package (Bioconductor software). Sequence Detection Systems 2.1 software was used to analyze the genotyping results.Data were represented as mean ± SEM. Data were compared using the $\chi 2$ test, the Fisher's exact test, the two-sided Student's t-test or one-way ANOVA where appropriate. In all cases, $p < 0.05$ was considered as statistical significance. All statistical tests were performed by the GraphPad Prism version 5.0 software (San Diego, CA, USA).The images were created by using PowerPoint 2016.

For manuscripts utilizing custom algorithms or software that are central to the research but not yet described in published literature, software must be made available to editors and reviewers. We strongly encourage code deposition in a community repository (e.g. GitHub). See the Nature Portfolio guidelines for submitting code & software for further information.

Data

Policy information about availability of data

All manuscripts must include a data availability statement. This statement should provide the following information, where applicable:

- Accession codes, unique identifiers, or web links for publicly available datasets
- A description of any restrictions on data availability
- For clinical datasets or third party data, please ensure that the statement adheres to our policy

The data that support the findings of this study are provided within the manuscript and Supplementary Information file. The RNA-seq data have been deposited in the NCBI public repository Sequence Read Archive under accession code SRP400184 https://www.ncbi.nlm.nih.gov/sra/?term=SRP400184. The metabolomics data have been deposited in the MetaboLights under accession code MTBLS8150 URL www.ebi.ac.uk/metabolights/MTBLS8150. Mouse mm10 reference genome used in this study is available from https://hgdownload.soe.ucsc.edu/goldenPath/mm10/bigZips/. Human hg19 reference genome used in this study is available from http://hgdownload.cse.ucsc.edu/goldenPath/hg19/bigZips/. Unique reagents used in this article are available from the corresponding authors upon reasonable request. The raw numbers for charts and graphs are available in the Source Data file whenever possible.

Field-specific reporting

Please select the one below that is the best fit for your research. If you are not sure, read the appropriate sections before making your selection.

[x] Life sciences   [ ] Behavioural & social sciences   [ ] Ecological, evolutionary & environmental sciences

For a reference copy of the document with all sections, see nature.com/documents/nr-reporting-summary-flat.pdf

Life sciences study design

All studies must disclose on these points even when the disclosure is negative.

Sample size	No statistical methods were used to predetermine the sample size. Sample size was determined based on previous experiments in the prior publications of our lab using similar methodologies [1, 2].<br><br>[1] Zhang J, Chen L, Wang F, et al. (2020) Extracellular HMGB1 exacerbates autoimmune progression and recurrence of type 1 diabetes by impairing regulatory T cell stability. Diabetologia 63(5): 987-1001. 10.1007/s00125-020-05105-8<br>[2] Yue T, Sun F, Wang F, et al. (2022) MBD2 acts as a repressor to maintain the homeostasis of the Th1 program in type 1 diabetes by regulating the STAT1-IFN-gamma axis. Cell Death Differ 29(1): 218-229. 10.1038/s41418-021-00852-6
Data exclusions	No data were excluded.
Replication	All data are the result of independently-repeated experiments with independent biological sample. Experiments were repeated independently at least twice with similar results in repeated experiments.
Randomization	Mice were randomized into the different groups.
Blinding	The investigators were blinded to group allocation during data collection and analysis.

Behavioural & social sciences study design

All studies must disclose on these points even when the disclosure is negative.

Study description	*Briefly describe the study type including whether data are quantitative, qualitative, or mixed-methods (e.g. qualitative cross-sectional, quantitative experimental, mixed-methods case study).*
Research sample	*State the research sample (e.g. Harvard university undergraduates, villagers in rural India) and provide relevant demographic information (e.g. age, sex) and indicate whether the sample is representative. Provide a rationale for the study sample chosen. For studies involving existing datasets, please describe the dataset and source.*
Sampling strategy	*Describe the sampling procedure (e.g. random, snowball, stratified, convenience). Describe the statistical methods that were used to predetermine sample size OR if no sample-size calculation was performed, describe how sample sizes were chosen and provide a rationale for why these sample sizes are sufficient. For qualitative data, please indicate whether data saturation was considered, and what criteria were used to decide that no further sampling was needed.*
Data collection	*Provide details about the data collection procedure, including the instruments or devices used to record the data (e.g. pen and paper, computer, eye tracker, video or audio equipment) whether anyone was present besides the participant(s) and the researcher, and whether the researcher was blind to experimental condition and/or the study hypothesis during data collection.*

Timing	*Indicate the start and stop dates of data collection. If there is a gap between collection periods, state the dates for each sample cohort.*
Data exclusions	*If no data were excluded from the analyses, state so OR if data were excluded, provide the exact number of exclusions and the rationale behind them, indicating whether exclusion criteria were pre-established.*
Non-participation	*State how many participants dropped out/declined participation and the reason(s) given OR provide response rate OR state that no participants dropped out/declined participation.*
Randomization	*If participants were not allocated into experimental groups, state so OR describe how participants were allocated to groups, and if allocation was not random, describe how covariates were controlled.*

Ecological, evolutionary & environmental sciences study design

All studies must disclose on these points even when the disclosure is negative.

Study description	*Briefly describe the study. For quantitative data include treatment factors and interactions, design structure (e.g. factorial, nested, hierarchical), nature and number of experimental units and replicates.*
Research sample	*Describe the research sample (e.g. a group of tagged Passer domesticus, all Stenocereus thurberi within Organ Pipe Cactus National Monument), and provide a rationale for the sample choice. When relevant, describe the organism taxa, source, sex, age range and any manipulations. State what population the sample is meant to represent when applicable. For studies involving existing datasets, describe the data and its source.*
Sampling strategy	*Note the sampling procedure. Describe the statistical methods that were used to predetermine sample size OR if no sample-size calculation was performed, describe how sample sizes were chosen and provide a rationale for why these sample sizes are sufficient.*
Data collection	*Describe the data collection procedure, including who recorded the data and how.*
Timing and spatial scale	*Indicate the start and stop dates of data collection, noting the frequency and periodicity of sampling and providing a rationale for these choices. If there is a gap between collection periods, state the dates for each sample cohort. Specify the spatial scale from which the data are taken*
Data exclusions	*If no data were excluded from the analyses, state so OR if data were excluded, describe the exclusions and the rationale behind them, indicating whether exclusion criteria were pre-established.*
Reproducibility	*Describe the measures taken to verify the reproducibility of experimental findings. For each experiment, note whether any attempts to repeat the experiment failed OR state that all attempts to repeat the experiment were successful.*
Randomization	*Describe how samples/organisms/participants were allocated into groups. If allocation was not random, describe how covariates were controlled. If this is not relevant to your study, explain why.*
Blinding	*Describe the extent of blinding used during data acquisition and analysis. If blinding was not possible, describe why OR explain why blinding was not relevant to your study.*

Did the study involve field work? ☐ Yes ☐ No

Field work, collection and transport

Field conditions	*Describe the study conditions for field work, providing relevant parameters (e.g. temperature, rainfall).*
Location	*State the location of the sampling or experiment, providing relevant parameters (e.g. latitude and longitude, elevation, water depth).*
Access & import/export	*Describe the efforts you have made to access habitats and to collect and import/export your samples in a responsible manner and in compliance with local, national and international laws, noting any permits that were obtained (give the name of the issuing authority, the date of issue, and any identifying information).*
Disturbance	*Describe any disturbance caused by the study and how it was minimized.*

Reporting for specific materials, systems and methods

We require information from authors about some types of materials, experimental systems and methods used in many studies. Here, indicate whether each material, system or method listed is relevant to your study. If you are not sure if a list item applies to your research, read the appropriate section before selecting a response.

Materials & experimental systems

n/a	Involved in the study
	☒ Antibodies
☒	Eukaryotic cell lines
☒	Palaeontology and archaeology
	☒ Animals and other organisms
	☒ Human research participants
☒	Clinical data
☒	Dual use research of concern

Methods

n/a	Involved in the study
☒	ChIP-seq
	☒ Flow cytometry
☒	MRI-based neuroimaging

Antibodies

Antibodies used

anti-cleaved caspase-3 (5A1E; 9664S; Cell Signaling Technology, Danvers, MA, USA, 1: 1000)
anti-Akt (9272S; Cell Signaling Technology, Danvers, MA, USA, 1: 1000)
anti-phospho-Akt (Ser473) (D9E; 4060S; Cell Signaling Technology, Danvers, MA, USA, 1: 1000)
anti-phospho-myosin light chain 2 (Ser19) (3675S; Cell Signaling Technology, Danvers, MA, USA, 1: 1000)
anti-p-LIMK-1/2 (Thr508/505) (Sc-28409-R; Santa Cruz Biotechnology, Santa Cruz, CA, USA, 1: 200)
anti-p-Cofilin (mSer 3) (Sc-21867-R; Santa Cruz Biotechnology, Santa Cruz, CA, USA, 1: 200)
anti-beta Actin (C4; Sc-47778; Santa Cruz Biotechnology, Santa Cruz, CA, USA, 1: 200)
anti-Myo9b (12432-1-AP; Proteintech, Wuhan, China, 1:1000)
anti-DYKDDDDK tag (20543-1-AP; Proteintech, Wuhan, China, 1:1000)
anti-PTEN (22034-1-AP; Proteintech, Wuhan, China, 1:1000)
anti-phospho-PTEN (Ser380) (AP0930; Abclonal, Wuhan, China, 1:1000)
anti-RhoA (67B9; 2117S; Cell Signaling Technology, Danvers, MA, USA, 1:1000)
anti-insulin (GB12334; servicebio, Wuhan, China, 1:100)
anti-CD11c (GB11059; servicebio, Wuhan, China, 1:100)
anti-CD3 (GB13014; servicebio, Wuhan, China, 1:100)
FITC anti-mouse CD11c (N418; 117306; Biolegend, San Diego, CA, USA, 1:200)
PE/Cy7 anti-mouse CD11c (N418; 117318; Biolegend, San Diego, CA, USA, 1:200)
Alexa Fluor® 647 anti-mouse I-Ad (39-10-8; 115010; Biolegend, San Diego, CA, USA, 1:200)
Brilliant Violet 421™ anti-mouse/human CD11b (M1/70; 101235; Biolegend, San Diego, CA, USA, 1:200)
Brilliant Violet 421™ anti-mouse CD4 (GK1.5; 100438; Biolegend, San Diego, CA, USA, 1:200)
FITC anti-mouse CD4 (GK1.5; 100406; Biolegend, San Diego, CA, USA, 1:200)
PerCP anti-mouse CD8a (53-6.7; 100731; Biolegend, San Diego, CA, USA, 1:200)
PE anti-mouse CD8a (53-6.7; 100708; Biolegend, San Diego, CA, USA, 1:200)
PE/Cy7 anti-mouse CD8a (53-6.7; 100722; Biolegend, San Diego, CA, USA, 1:200)
FITC anti-human HLA-DR (L243; 307604; Biolegend, San Diego, CA, USA, 1:200)
APC anti-mouse CD62L (MEL-14; 104412; Biolegend, San Diego, CA, USA, 1:200)
PE anti-mouse CD62L (MEL-14; 104408; Biolegend, San Diego, CA, USA, 1:200)
APC anti-mouse/human CD44 (IM7; 103012; Biolegend, San Diego, CA, USA, 1:200)
PE anti-mouse/human CD44 (IM7; 103008; Biolegend, San Diego, CA, USA, 1:200)
Brilliant Violet 421™ anti-mouse IFN-γ (XMG1.2; 505829; Biolegend, San Diego, CA, USA, 1:200)
PE/Cy7 anti-mouse IFN-γ (XMG1.2; 505826; Biolegend, San Diego, CA, USA, 1:200)
Brilliant Violet 421™ anti-mouse IL-17A (TC11-18H10.1; 506926; Biolegend, San Diego, CA, USA, 1:200)
Alexa Fluor® 647 anti-mouse/rat/human FOXP3 (150D; 320014; Biolegend, San Diego, CA, USA, 1:200)
PE/Cy7 anti-mouse CD86 (GL-1; 105014; Biolegend, San Diego, CA, USA, 1:200)
FITC anti-mouse H-2Kd (SF1-1.1; 116605; Biolegend, San Diego, CA, USA, 1:200)
Alexa Fluor® 488 anti-DYKDDDDK tag antibody (L5; 637317; Biolegend, San Diego, CA, USA, 1:200)
PE hamster anti-mouse CD80 (16-10A1; 553769, BD Bioscience, San Diego, CA, USA, 1:200)

Validation

anti-cleaved caspase-3 (9664s) https://www.cellsignal.cn/products/primary-antibodies/cleaved-caspase-3-asp175-5a1e-rabbit-mab/9664?site-search-type=Products&N=4294956287&Ntt=9664s&fromPage=plp&_requestid=1550276
anti-Akt (9272S) https://www.cellsignal.cn/products/primary-antibodies/akt-antibody/9272?site-search-type=Products&N=4294956287&Ntt=9272s&fromPage=plp&_requestid=7170227
anti-phospho-Akt (Ser473) (4060S) https://www.cellsignal.cn/products/primary-antibodies/phospho-akt-ser473-d9e-xp-rabbit-mab/4060?site-search-type=Products&N=4294956287&Ntt=4060s&fromPage=plp&_requestid=7170348
anti-phospho-myosin light chain 2 (Ser19) (3675S) https://www.cellsignal.cn/products/primary-antibodies/phospho-myosin-light-chain-2-ser19-mouse-mab/3675?site-search-type=Products&N=4294956287&Ntt=3675s&fromPage=plp&_requestid=7170445
anti-p-LIMK-1/2 (Thr508/505) (Sc-28409-R) https://datasheets.scbt.com/sc-28409.pdf
anti-p-Cofilin (mSer 3) (Sc-21867-R)https://www.scbt.com/p/p-cofilin-antibody-mser-3?requestFrom=search
anti-beta Actin (Sc-47778) https://www.scbt.com/p/beta-actin-antibody-c4?requestFrom=search
anti-Myo9b (12432-1-AP) https://www.ptglab.com/products/MYO9B-Antibody-12432-1-AP.htm
anti-DYKDDDDK tag (20543-1-AP) https://www.ptglab.com/products/Flag-Tag-Antibody-20543-1-AP.htm
anti-PTEN (22034-1-AP) https://www.ptglab.com/products/PTEN-Antibody-22034-1-AP.htm
anti-phospho-PTEN (Ser380) (AP0930) https://abclonal.com.cn/catalog/AP0930

anti-RhoA (2117S) https://www.cellsignal.cn/products/primary-antibodies/rhoa-67b9-rabbit-mab/2117?site-search-type=Products&N=4294956287&Ntt=2117s&fromPage=plp&_requestid=7172063
anti-insulin (GB12334) https://www.servicebio.cn/goodsdetail?id=6012
anti-CD11c (GB11059) https://www.servicebio.cn/goodsdetail?id=1340
anti-CD3 (GB13014) https://www.servicebio.cn/goodsdetail?id=633
FITC anti-mouse CD11c (117306) https://www.biolegend.com/en-us/products/fitc-anti-mouse-cd11c-antibody-1815
APC anti-mouse CD11c (117310) https://www.biolegend.com/en-us/products/apc-anti-mouse-cd11c-antibody-1813
PE/Cy7 anti-mouse CD11c (117318) https://www.biolegend.com/en-us/products/pe-cyanine7-anti-mouse-cd11c-antibody-3086
Alexa Fluor® 647 anti-mouse I-Ad (115010) https://www.biolegend.com/en-us/products/alexa-fluor-647-anti-mouse-i-ad-antibody-3228
Brilliant Violet 421™ anti-mouse/human CD11b (101235) https://www.biolegend.com/en-us/products/brilliant-violet-421-anti-mouse-human-cd11b-antibody-7163
Brilliant Violet 421™ anti-mouse CD4 (100438) https://www.biolegend.com/en-us/products/brilliant-violet-421-anti-mouse-cd4-antibody-7142
FITC anti-mouse CD4 (100406) https://www.biolegend.com/en-us/products/fitc-anti-mouse-cd4-antibody-248
PerCP anti-mouse CD8a (100731) https://www.biolegend.com/en-us/products/percp-anti-mouse-cd8a-antibody-4256
PE anti-mouse CD8a (100708) https://www.biolegend.com/en-us/products/pe-anti-mouse-cd8a-antibody-155
PE/Cy7 anti-mouse CD8a (100722) https://www.biolegend.com/en-us/products/pe-cyanine7-anti-mouse-cd8a-antibody-1906
FITC anti-human HLA-DR (307604) https://www.biolegend.com/en-us/products/fitc-anti-human-hla-dr-antibody-788
APC anti-mouse CD62L (104412) https://www.biolegend.com/en-us/products/apc-anti-mouse-cd62l-antibody-381
PE anti-mouse CD62L (104408) https://www.biolegend.com/en-us/products/pe-anti-mouse-cd62l-antibody-386
APC anti-mouse/human CD44 (103012) https://www.biolegend.com/en-us/products/apc-anti-mouse-human-cd44-antibody-312
PE anti-mouse/human CD44 (103008) https://www.biolegend.com/en-us/products/pe-anti-mouse-human-cd44-antibody-2206
Brilliant Violet 421™ anti-mouse IFN-γ (505829) https://www.biolegend.com/en-us/products/brilliant-violet-421-anti-mouse-ifn-gamma-antibody-7154
PE/Cy7 anti-mouse IFN-γ (505826) https://www.biolegend.com/en-us/products/pe-cyanine7-anti-mouse-ifn-gamma-antibody-5865
Brilliant Violet 421™ anti-mouse IL-17A (506926) https://www.biolegend.com/en-us/products/brilliant-violet-421-anti-mouse-il-17a-antibody-7223
Alexa Fluor® 647 anti-mouse/rat/human FOXP3 (320014) https://www.biolegend.com/en-us/products/alexa-fluor-647-anti-mouse-rat-human-foxp3-antibody-2892
PE/Cy7 anti-mouse CD86 (105014) https://www.biolegend.com/en-us/products/pe-cyanine7-anti-mouse-cd86-antibody-3046
FITC anti-mouse H-2Kd (116605) https://www.biolegend.com/en-us/products/fitc-anti-mouse-h-2kd-antibody-1860
Alexa Fluor® 488 anti-DYKDDDDK tag antibody (637317) https://www.biolegend.com/en-us/products/alexa-fluor-488-anti-dykddddk-tag-15653
PE hamster anti-mouse CD80 (553769) https://www.bdbiosciences.com/zh-cn/products/reagents/flow-cytometry-reagents/research-reagents/single-color-antibodies-ruo/pe-hamster-anti-mouse-cd80.553769

Eukaryotic cell lines

Policy information about cell lines

Cell line source(s) | *State the source of each cell line used.*

Authentication | *Describe the authentication procedures for each cell line used OR declare that none of the cell lines used were authenticated.*

Mycoplasma contamination | *Confirm that all cell lines tested negative for mycoplasma contamination OR describe the results of the testing for mycoplasma contamination OR declare that the cell lines were not tested for mycoplasma contamination.*

Commonly misidentified lines (See ICLAC register) | *Name any commonly misidentified cell lines used in the study and provide a rationale for their use.*

Palaeontology and Archaeology

Specimen provenance | *Provide provenance information for specimens and describe permits that were obtained for the work (including the name of the issuing authority, the date of issue, and any identifying information). Permits should encompass collection and, where applicable, export.*

Specimen deposition | *Indicate where the specimens have been deposited to permit free access by other researchers.*

Dating methods | *If new dates are provided, describe how they were obtained (e.g. collection, storage, sample pretreatment and measurement), where they were obtained (i.e. lab name), the calibration program and the protocol for quality assurance OR state that no new dates are provided.*

☐ Tick this box to confirm that the raw and calibrated dates are available in the paper or in Supplementary Information.

Ethics oversight | *Identify the organization(s) that approved or provided guidance on the study protocol, OR state that no ethical approval or guidance was required and explain why not.*

Note that full information on the approval of the study protocol must also be provided in the manuscript.

Animals and other organisms

Policy information about studies involving animals; ARRIVE guidelines recommended for reporting animal research

Laboratory animals	NOD/ShiLtJ and ALR/Lt mice were used. Mouse models generated for the study include CD11c-Cre Myo9bflox/flox NOD mice and ALR Myo9b KI NOD mice. All mice were maintained under specific pathogen-free conditions with 20-24°C ambient temperature, 45-65% humidity, and a 12/12 h light/dark cycle. Female mice were monitored for blood glucose after 10 weeks of age or adoptive transfer of diabetogenic CD4+ T cells. Mice of both sexes (5- to 6-week-old, 10- to 12-week-old, or 16-week-old) were used for other experiments.
Wild animals	No wild animals were used in the study.
Field-collected samples	The study did not involve samples collected from the field.
Ethics oversight	All animal experiments were performed in accordance with the National Institutes of Health (NIH) guidelines. The study was approved by the Tongji Hospital Animal Care and Use Committee (TJH-201903020).

Note that full information on the approval of the study protocol must also be provided in the manuscript.

Human research participants

Policy information about studies involving human research participants

Population characteristics	The study consisted of 1,298 unrelated T1D patients and 2,936 healthy controls recruited from Tongji Hospital, the Second Xiangya Hospital of Central South University, and the Third Affiliated Hospital of Sun Yat-sen University. Clinical characteristics of the study population were provided in Table S4.
Recruitment	The clinical diagnosis for T1D were according to the World Health Organization (WHO) criteria, and only patients with the presence of at least one autoantibody against islet (IAA, ZnT8, or GADA) were included as T1D cases. Healthy controls, who did not have overt autoimmune diseases or diabetes mellitus or any other chronic diseases, were enrolled from the same geographical regions.
Ethics oversight	Informed consent was obtained from all of the individuals included. The study was performed in accordance with the Declaration of Helsinki and was approved by the Institutional Review Board (IRB) of Tongji Hospital (TJ-IRB20160602).

Note that full information on the approval of the study protocol must also be provided in the manuscript.

Clinical data

Policy information about clinical studies

All manuscripts should comply with the ICMJEguidelines for publication of clinical research and a completedCONSORT checklist must be included with all submissions.

Clinical trial registration	*Provide the trial registration number from ClinicalTrials.gov or an equivalent agency.*
Study protocol	*Note where the full trial protocol can be accessed OR if not available, explain why.*
Data collection	*Describe the settings and locales of data collection, noting the time periods of recruitment and data collection.*
Outcomes	*Describe how you pre-defined primary and secondary outcome measures and how you assessed these measures.*

Dual use research of concern

Policy information about dual use research of concern

Hazards

Could the accidental, deliberate or reckless misuse of agents or technologies generated in the work, or the application of information presented in the manuscript, pose a threat to:

No	Yes	
☐	☐	Public health
☐	☐	National security
☐	☐	Crops and/or livestock
☐	☐	Ecosystems
☐	☐	Any other significant area

Experiments of concern

Does the work involve any of these experiments of concern:

No	Yes	
☐	☐	Demonstrate how to render a vaccine ineffective
☐	☐	Confer resistance to therapeutically useful antibiotics or antiviral agents
☐	☐	Enhance the virulence of a pathogen or render a nonpathogen virulent
☐	☐	Increase transmissibility of a pathogen
☐	☐	Alter the host range of a pathogen
☐	☐	Enable evasion of diagnostic/detection modalities
☐	☐	Enable the weaponization of a biological agent or toxin
☐	☐	Any other potentially harmful combination of experiments and agents

ChIP-seq

Data deposition

☐ Confirm that both raw and final processed data have been deposited in a public database such as GEO.

☐ Confirm that you have deposited or provided access to graph files (e.g. BED files) for the called peaks.

Data access links<br>*May remain private before publication.*	*For "Initial submission" or "Revised version" documents, provide reviewer access links. For your "Final submission" document, provide a link to the deposited data.*
Files in database submission	*Provide a list of all files available in the database submission.*
Genome browser session<br>(e.g. UCSC)	*Provide a link to an anonymized genome browser session for "Initial submission" and "Revised version" documents only, to enable peer review. Write "no longer applicable" for "Final submission" documents.*

Methodology

Replicates	*Describe the experimental replicates, specifying number, type and replicate agreement.*
Sequencing depth	*Describe the sequencing depth for each experiment, providing the total number of reads, uniquely mapped reads, length of reads and whether they were paired- or single-end.*
Antibodies	*Describe the antibodies used for the ChIP-seq experiments; as applicable, provide supplier name, catalog number, clone name, and lot number.*
Peak calling parameters	*Specify the command line program and parameters used for read mapping and peak calling, including the ChIP, control and index files used.*
Data quality	*Describe the methods used to ensure data quality in full detail, including how many peaks are at FDR 5% and above 5-fold enrichment.*
Software	*Describe the software used to collect and analyze the ChIP-seq data. For custom code that has been deposited into a community repository, provide accession details.*

Flow Cytometry

Plots

Confirm that:

☒ The axis labels state the marker and fluorochrome used (e.g. CD4-FITC).

☒ The axis scales are clearly visible. Include numbers along axes only for bottom left plot of group (a 'group' is an analysis of identical markers).

☒ All plots are contour plots with outliers or pseudocolor plots.

☒ A numerical value for number of cells or percentage (with statistics) is provided.

Methodology

Sample preparation	Cells were retrieved from spleen and pancreatic LNs by meshing the organs through a 70-µm strainer with a syringe plunger. The pancreas was inflated via the bile duct with digestion buffer containing 0.5mg/ml collagenase P. The distended pancreas was removed and digested at 38℃ for 6 min. To isolate cells from the colonic lamina propria, colons were first cleaned by flushing the luminal content out with PBS, defatted, opened up longitudinally, and diced into 1-2-cm pieces. Tissues were then incubated in 2 ml IEL solution (1× PBS with 2% FBS, 10 mM Hepes buffer, 1% penicillin/streptomycin, and 1% L-

glutamine plus 1 mM EDTA and 1 mM DTT added immediately before use) for 15 min at 37°C with vigorous shaking to remove the epithelial fraction. Tissues were then retrieved, washed extensively, and digested in 2 ml LPL solution (1× RPMI 1640 with 2% FBS, 10 mM Hepes buffer, 1% penicillin/streptomycin, 1% L-glutamine, 2mg/ml collagenase IV, and 1 U/ml DNase I) for 30 min at 37°C with vigorous shaking. The digested samples were passed through a 70-µm strainer, pelleted at 300 g for 5 min, and washed extensively. Salivary gland, lung, kidney, liver, and heart were digested in the similar fashion as the lamina propria fraction of the colons for 30 min. Erythrocytes in the samples were lysed with ammonium chloride potassium lysis buffer. For cell surface staining, single-cell suspension was incubated with indicated antibodies for 30 min on ice. Intracellular staining was performed using the Transcription Factor Buffer Set (562574; BD Biosciences, San Diego, CA, USA) with the antibody cocktails. For intracellular cytokine staining, cells were stimulated with Cell Activation Cocktail (423303; Biolegend, San Diego, CA, USA) for 4-6 h. Antibodies used are listed above.

Instrument

MACS Quant Analyzer10 (Miltenyi Biotec, Germany)

Software

FlowJo software v10.5.3

Cell population abundance

Naïve T cells were isolated from splenocytes of 5-week-old BDC2.5 NOD mice with a MojoSort™ Mouse CD4 Naïve T Cell Isolation Kit (480040; Biolegend, San Diego, CA, USA) according to the manufacturer's instructions. The purity of isolated naive T cells was determined by FACS.

Gating strategy

Cells were gated by FSC/SSC gates to select single cells. After that the detail gating strategy was showed as follows:
mouse effector T cells: CD4+CD44highCD62Llo
mouse naïve T cells: CD4+CD44loCD62Lhigh
mouse Th1 cells: CD4+ IFN-γ+
mouse Tc1 cells: CD8+ IFN-γ+
mouse Th17 cells: CD4+IL-17A+
mouse Treg cells: CD4+Foxp3+
mouse dendritic cells: CD11c+MHCII+
mouse cDC1: CD11c+MHCII+CD8a+
mouse cDC2: CD11c+MHCII+CD11b+

☒ Tick this box to confirm that a figure exemplifying the gating strategy is provided in the Supplementary Information.

Magnetic resonance imaging

Experimental design

Design type

Indicate task or resting state; event-related or block design.

Design specifications

Specify the number of blocks, trials or experimental units per session and/or subject, and specify the length of each trial or block (if trials are blocked) and interval between trials.

Behavioral performance measures

State number and/or type of variables recorded (e.g. correct button press, response time) and what statistics were used to establish that the subjects were performing the task as expected (e.g. mean, range, and/or standard deviation across subjects).

Acquisition

Imaging type(s)

Specify: functional, structural, diffusion, perfusion.

Field strength

Specify in Tesla

Sequence & imaging parameters

Specify the pulse sequence type (gradient echo, spin echo, etc.), imaging type (EPI, spiral, etc.), field of view, matrix size, slice thickness, orientation and TE/TR/flip angle.

Area of acquisition

State whether a whole brain scan was used OR define the area of acquisition, describing how the region was determined.

Diffusion MRI ☐ Used ☐ Not used

Preprocessing

Preprocessing software

Provide detail on software version and revision number and on specific parameters (model/functions, brain extraction, segmentation, smoothing kernel size, etc.).

Normalization

If data were normalized/standardized, describe the approach(es): specify linear or non-linear and define image types used for transformation OR indicate that data were not normalized and explain rationale for lack of normalization.

Normalization template

Describe the template used for normalization/transformation, specifying subject space or group standardized space (e.g. original Talairach, MNI305, ICBM152) OR indicate that the data were not normalized.

Noise and artifact removal

Describe your procedure(s) for artifact and structured noise removal, specifying motion parameters, tissue signals and physiological signals (heart rate, respiration).

Volume censoring | *Define your software and/or method and criteria for volume censoring, and state the extent of such censoring.*

Statistical modeling & inference

Model type and settings | *Specify type (mass univariate, multivariate, RSA, predictive, etc.) and describe essential details of the model at the first and second levels (e.g. fixed, random or mixed effects; drift or auto-correlation).*

Effect(s) tested | *Define precise effect in terms of the task or stimulus conditions instead of psychological concepts and indicate whether ANOVA or factorial designs were used.*

Specify type of analysis: ☐ Whole brain ☐ ROI-based ☐ Both

Statistic type for inference (See Eklund et al. 2016) | *Specify voxel-wise or cluster-wise and report all relevant parameters for cluster-wise methods.*

Correction | *Describe the type of correction and how it is obtained for multiple comparisons (e.g. FWE, FDR, permutation or Monte Carlo).*

Models & analysis

n/a	Involved in the study
☐	☐ Functional and/or effective connectivity
☐	☐ Graph analysis
☐	☐ Multivariate modeling or predictive analysis

Functional and/or effective connectivity | *Report the measures of dependence used and the model details (e.g. Pearson correlation, partial correlation, mutual information).*

Graph analysis | *Report the dependent variable and connectivity measure, specifying weighted graph or binarized graph, subject- or group-level, and the global and/or node summaries used (e.g. clustering coefficient, efficiency, etc.).*

Multivariate modeling and predictive analysis | *Specify independent variables, features extraction and dimension reduction, model, training and evaluation metrics.*
